# New Books

**Published:** 2005-05

**An Introduction to Human Molecular Genetics: Mechanisms of Inherited Diseases, 2nd ed.**

Jack K. Pasternak

Hoboken, NJ:John Wiley & Sons, 2005. 656 pp. ISBN: 0-471-47426-6, $83.95

**CCN Proteins: A New Family of Cell Growth and Differentiation Regulators**

Bernard Perbal, Masaharu Takigawa

Hackensack, NJ:World Scientific Publishing, 2005. 250 pp. ISBN: 1-86094-552-X, $80

**Computation in Cells and Tissues: Perspectives and Tools of Thought**

R. Paton, H. Bolouri, M. Holcombe, J.H. Parish, R. Tateson, eds.

New York:Springer-Verlag, 2004. 343 pp. ISBN: 3-540-00358-4, $69.95

**Data Analysis and Visualization in Genomics and Proteomics**

Francisco Azuaje, Joaquin Dopazo, eds.

Hoboken, NJ:John Wiley & Sons, 2005. 284 pp. ISBN: 0-470-09439-7, $150

**Energy in the 21st Century**

John R. Fanchi

Hackensack, NJ:World Scientific Publishing, 2005. 256 pp. ISBN: 981-256-185-4, $52

**Environmental Economics for Non-Economists: Techniques and Policies for Sustainable Development, 2nd ed.**

John Asafu-Adjaye

Hackensack, NJ:World Scientific Publishing, 2005. 392 pp. ISBN: 981-256-123-4, $58

**Evolution in Four Dimensions: Genetic, Epigenetic, Behavioral, and Symbolic Variation in the History of Life**

Eva Jablonka, Marion J. Lamb

Cambridge, MA:MIT Press, 2005. 472 pp. ISBN: 0-262-10107-6, $34.95

**Implications of Nanotechnology for Environmental Health Research**

Lynn Goldman, Christine Coussens, eds.

Washington, DC:National Academies Press, 2005. 70 pp. ISBN: 0-309-09577-8, $18

**Intelligent Bioinformatics: The Application of Artificial Intelligence Techniques to Bioinformatics Problems**

Edward Keedwell, Ajit Narayanan

Hoboken, NJ:John Wiley & Sons, 2005. 256 pp. ISBN: 0-470-02175-6, $110

**Medical Biomethods Handbook**

John M. Walker, Ralph Rapley

Totowa, NJ:Humana Press, 2005. 656 pp. ISBN: 1-58829-288-6, $150

**Molecular and Cellular Signaling**

Martin Beckerman

New York:Springer-Verlag, 2005. 450 pp. ISBN: 0-387-22130-1, $99

**Molecular Models of Life: Philosophical Papers on Molecular Biology**

Sahotra Sarkar

Cambridge, MA:MIT Press, 2005. 352 pp. ISBN: 0-262-19512-7, $38

**Mouse Phenotypes: A Handbook of Mutation Analysis**

Virginia E. Papaioannou

Woodbury, NY:Cold Spring Harbor Laboratory Press, 2005. 235 pp. ISBN: 0-87969-640-0, $80

**Protein Nanotechnology: Protocols, Instrumentation, and Applications**

Tuan Vo-Dinh

Totowa, NJ:Humana Press, 2005. 480 pp. ISBN: 1-58829-310-6, $115

**Selective Sweep**

Dmitry Nurminsky

New York:Springer-Verlag, 2005. 130 pp. ISBN: 0-306-48235-5, $129

**The Global Genome: Biotechnology, Politics, and Culture**

Eugene Thacker

Cambridge, MA:MIT Press, 2005. 464 pp. ISBN: 0-262-20155-0, $39.95

**The Inside Story: DNA to RNA to Protein**

Jan Witkowski, ed.

Woodbury, NY:Cold Spring Harbor Laboratory Press, 2005. 220 pp. ISBN: 0-87969-750-4, $45

**The Proteomics Protocols Handbook**

John M. Walker

Totowa, NJ:Humana Press, 2005. 1,016 pp. ISBN: 1-58829-343-2, $175

**XML for Bioinformatics**

Ethan Cerami

New York:Springer-Verlag, 2005. 304 pp. ISBN: 0-387-23028-9, $69.95

